# Selective phosphoinositide 3-kinase inhibitors and implication in diabetic retinopathy as pharmacological tools

**DOI:** 10.3389/fphar.2026.1744181

**Published:** 2026-03-13

**Authors:** Carmela Bonaccorso, Francesca Lazzara, Isabel La Rosa, Cristina Munzone, Alessandro Grasso, Chiara B. M. Platania, Claudio Bucolo

**Affiliations:** 1 Department of Chemical Sciences, University of Catania, Catania, Italy; 2 Department of Biomedical and Biotechnological Sciences, University of Catania, Catania, Italy; 3 Center for Research in Ocular Pharmacology-CERFO, University of Catania, Catania, Italy; 4 Department of Medicine and Surgery, “Kore” University of Enna, Enna, Italy

**Keywords:** diabetic retinopathy, PI3K inhibitor, quantitative structure activity relationship (QSAR), repurposing, retina

## Abstract

Phosphoinositide 3-kinases (PI3Ks) are ubiquitous enzymes, that regulate different cellular functions, most involved in pathogenesis and progression of several oncological diseases. Indeed, some PI3K inhibitors have been approved for blood cancers, such as lymphoma. Interestingly, leniolisib, a selective PI3Kδ kinase inhibitor, has been approved for the rare disease Activated Phosphoinositide 3-kinase Delta Syndrome (APDS). Activation of PI3K/AKT signaling is downstream to VEGF-A pro-angiogenic signaling, detrimental in diabetic retinopathy progression, a microvascular complication of diabetes mellitus. Recently, a report evidenced that inhibition of class IA PI3K (PI3Kδ) delivered beneficial effects in an *in-vivo* model of diabetic retinopathy. We hereby explored the implication of PI3K signaling in diabetic retinopathy. Moreover, we reviewed the current literature to highlight molecular features of class I PI3K selective inhibitors, to further guide the design of novel selective and safe drugs targeting PI3Kδ, for management of diabetic retinopathy or other retinal proliferative diseases.

## 1 Introduction

**FIGURE 1:**
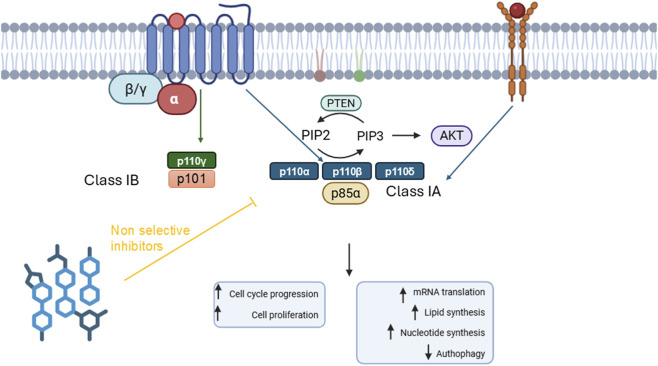
PI3K class IA and class IB signaling pathway. Class IA PI3K are activated by GPCRs and receptor tyrosine kinases. While class IB PI3K is activated only by GPCR signaling. PI3Ks generate PtdIns (3,4,5) P_3_ from PtdIns (4,5) P_2_, then modulating several cellular functions.

Phosphoinositide 3-kinases (PI3Ks) represent ubiquitous lipid kinases involved in many processes including the cell cycle, growth and response to external signals and stimulation. These enzymes phosphorylate the inositol ring of phosphatidyl inositol-4,5-biphosphate [PtdIns (4,5) P_2_] at the three-position (-OH) (Martelli et al., 2010), leading to phosphoinositide-triphosphates (PIP3), a second messenger (Vanhaesebroeck et al., 2005). Different classes of PI3Ks are activated by different upstream signaling mediated by GPCRs, receptor tyrosine kinases (RTK) (Figure 1), and Ras pathway.

The PIP3 recruits AKT to membrane, by binding to its N-terminal pleckstrin homology (PH) domain, thereby AKT is activated through phosphorylation by PDK1 at Thr308 and by mTORc2 at Ser473 (Chu et al., 2020). PI3K/PIP3 signal regulation is mediated by the tumor suppressor and phosphates PTEN, which catalyze the conversion of PIP3 to PIP2. PI3K/Akt/mTOR signaling pathway regulates several physiological processes, and when dysregulated contributes to both tumorigenesis and therapy resistance (Noorolyai et al., 2019).

Eight isoforms of PI3Ks have been classified and investigated in mammals, and these have been divided into three classes, class I, III and III. Class I PI3Ks are intersections of several intracellular signals, because they integrate a variety of pathways activated by growth factors and their receptor signaling (Safaroghli-Azar et al., 2023). Class I consists of four members, heterodimeric enzymes that phosphorylate PtdIns (4,5) P_2_ by turning it into PIP3 (Figure 1). Structurally, PI3K class I enzymes consist of a catalytic subunit, indicated as p110, and a subunit with a regulator/adaptor function called p85 (or p55), which anchors the plasma membrane through the interaction of the SH2 domains with phosphotyrosine residues of activated receptors (Fox et al., 2020). Furthermore, class I is further divided into two subclasses, IA and IB. Class IA PI3K is a heterodimer including the catalytic subunit p110α (110 kDa), and a regulatory subunit weighing p85α (85 kDa). There are five different isoforms of the regulatory subunit as result of alternative splicing: p85α, p85β, p55α, p50α and p55γ, but here the term p85 is used generically to refer to all Class IA PI3K regulatory subunits when the isoform is otherwise not stated (Fox et al., 2020).

**FIGURE 2:**
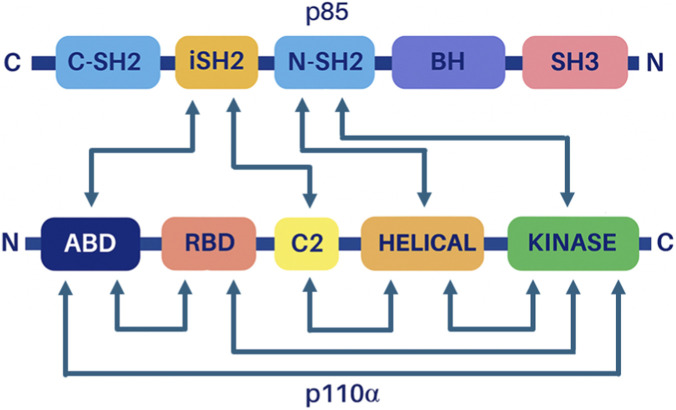
PI3K heterodimer domains. Interaction map between domains of the regulatory subunit (p85) and the catalytic subunit (p110). Adapted from (Zhao and Vogt, 2008).

The p110 subunit (Figure 2) is encoded by three different genes (p110α, p110β, p110δ). This subunit is characterized by several structural domains (ABD, RBD, C2, helical, kinase). Both ABD and C2 domains interact with intern-SH2 domain of p85 subunit (Figure 2). The C-terminal of p85 includes two SH2 domains, the C-terminal SH2 (C-SH2) and N-terminal SH2 (N-SH2) domains, separated by an inter-SH2 domain (iSH2) (Zhao and Vogt, 2008).

Activation of Class IA PI3Ks occurs upon binding of SH2 domains of p85 regulatory subunit to phosphotyrosines of the activated receptor tyrosine kinases, promoting not only activation of p110 catalytic subunit but also its recruitment at cell membrane (Fox et al., 2020).

Up to now, it has been proven that Class IA PI3Kβ can be activated also by Gβγ heterodimer upon GPCR activation (Dbouk et al., 2012; Wijayaratna et al., 2023).

The second subclass of PI3K class I is the IB class kinase, namely, PI3Kγ, which is activated exclusively by GPCRs (Rathinaswamy et al., 2023). The catalytic subunit p110γ, encoded by the PIK3CG, binds to p101 (encoded by PIK3R5) or p84 (encoded by PIK3R6), which is the regulatory subunit. This catalytic subunit is expressed in leukocytes where it is activated by Gβγ heterodimer, an interaction mediated mainly by the p101 regulatory subunit (Hawkins et al., 2010). In 2013, it has been proved that both regulatory (p101) and catalytic (p110) subunits of PI3Kγ interact with Gβγ heterodimer, that in turn promote activation of the PI3K kinase upon exposure to lipid membrane of a specific helical domain of p110γ (Vadas et al., 2013).

Class II PI3Ks consists of three members (PI3KC2 α, β, γ), C2 indicates the presence of a carboxy-terminal domain that preferentially binds to hosphatidylinositol-4,5-bisphosphate [PtdIns(4,5)*P*_2_]. In fact, these kinases use PI and PI(4)P as substrates. Class II enzymes are mainly bound to cellular membrane and are activated by membrane receptors such as RTKs and integrins (Gulluni et al., 2019).

Finally, class III includes a catalytic subunit, highly conserved in mammals, the vacuolar protein sorting 34 (VPS34p) responsible to produce PIP3 and involved in protein trafficking through lysosomes. The adaptor subunit (Vps15 or p150) is essential for Vps34 stability and activity (Panaretou et al., 1997). Vps34 regulates endosomal trafficking, autophagy, phagocytosis and nutrient-sensing signaling.

## 2 Mutations of class I PI3K genes

Mutations in the three genes - PIK3CA, PIK3CB, PIK3CD - encoding for the p110 subunit, have been associated to the development of solid cancers (Samuels et al., 2004; Madsen et al., 2018). Involvement of PI3K hyperactivation in cancers has burst the research focused on development of inhibitors of these ubiquitous lipid kinases (Tankova et al., 2022).

Furthermore, a rare immunodeficiency disease, the activated phosphoinositide kinase (PI3Kδ) syndrome (APDS) is characterized in the 75% of cases by a gain of function (GOF) mutation of p110δ gene (PI3KCD) and in the 25% of cases by loss of function (LOF) mutation of p85α gene (PI3KR1). APDS is characterized by the overactivation of PI3K in immune cells, which specifically express the mutated PI3Kδ, indeed recently, the Food and Drug Administration (FDA) approved leniolisib, a selective PI3Kδ inhibitor, as orphan drug for the treatment of APDS (Tommasini et al., 2024).

Class IB PI3K gain of function mutations have been also associated to tumorigenesis, such as the R1021C mutation, while p110γ loss of function mutation in the C-terminal domain (R1021P, N1085S) have been identified in patients with immunodeficiencies. These two mutations have been found to influence the structure and activity of PI3Kγ, along with binding, potency and mechanism of action of PI3Kγ inhibitors (Rathinaswamy et al., 2021).

## 3 Diabetic retinopathy and PI3K signaling

Diabetic retinopathy (DR) is classified into non-proliferative diabetic retinopathy (NPDR) and proliferative diabetic retinopathy (PDR), the latter characterized by retinal neovascularization and diabetic edema (Yang et al., 2022). The pathogenesis of DR is complex, although hyperglycemia constitutes the main factor triggering a series of reactions including increased production of catabolic products, glycosylation, polyol metabolism activity and altered signal transduction mechanisms (Wong et al., 2016).

Damage of retinal microvessels, that constitute the inner blood retinal barrier (iBRB), involves dysfunction and death of endothelial cells and pericytes, that in turn leads to an unbalanced blood flow, then resulting in hypoxic microenvironment (Lazzara et al., 2020). Hypoxia activates the transcription of pro-angiogenic factors, such as vascular endothelial growth factor (VEGF-A) and PlGF (Lazzara et al., 2019; 2020; Bonfiglio et al., 2020). Retinal angiogenesis mediated by pro-angiogenic factors, breakdown of blood-retinal barrier (BRB) and increased retinal permeability are responsible for diabetic macular edema (DME), the sight-threatening complication of DR. Currently approved drugs for the management of diabetic macular edema are anti-VEGF agents (first line) or glucocorticoids (second line or adjuvant treatments) that are delivered through invasive and costly intravitreal injections (Bucolo et al., 2018). Thereby, current available treatments for management of DR, are pan VEGF-A signaling inhibitors, addressing downstream the pathological mechanisms that cause diabetic retinopathy progression, but these therapies are associated to treatment resistance (Sharma et al., 2023), and to photoreceptor damage (Kurihara et al., 2012; Quaggin, 2012). Indeed, there is an urgent unmet medical need in the treatment of diabetic retinopathy, i.e., new pharmacological targets and tools.

An old, but comprehensive review, reported a strong link between diabetes mellitus and PI3K signaling, besides the direct connection between insulin receptor (IR) signaling and PI3Ks (Braccini et al., 2009). Specifically, insulin receptor substrates (IRS), after binding to active IR, are phosphorylated and became docking sites for p85 subunit of PI3Ks, promoting p110 activation and recruitment at cell membrane (Braccini et al., 2009).

Heterozygous loss of p110α or p110β had no effects on glucose metabolism in mice. On the contrary, mice with double heterozygous loss of p110α and p110β were found to be slightly glucose intolerant and showed hyperinsulinemia (Brachmann et al., 2005). A rare missense mutation in p85α (R649W), class I regulatory subunit, found in SHORT (Short stature, Hyperextensibility/Hernia, Ocular depression, Rieger anomaly, and Teething delay) patients, has been studied also in mice. Specifically, despite the lower level of adiposity, the high fat diet (HFD) induced in mutated mice hyperglycemia and insulin resistance in *Pik3r1* R649W knock-in mice, with levels higher than wild type mice fed with HFD (Solheim et al., 2018). Interestingly, PI3Kδ overexpression has been found in leucocytes of patients with gestational diabetes mellitus (Wójcik et al., 2014). Although PI3K/AKT signaling is a crossroad of several signaling pathways involved diabetes mellitus (Table 1) and thereby in diabetes complications pathogenesis and progression, such as DR, few reports suggest that PI3K inhibition would be a therapeutic strategy for treatment of DR as microvascular complication of diabetes (Taheri et al., 2024).

**TABLE 1:** Involvement of PI3K isoforms in diabetes and in diabetic retinopathy.

PI3K isoforms	Metabolism, diabetes and DR implications	References
Class IA (mutation on p85)	Hyperglycemia and insulin resistance	Solheim et al. (2018)
PI3Kα and PI3Kβ	Glucose intolerance and hyperinsulinemia	Brachmann et al. (2005)
PI3Kα	Angiogenesis and endothelial cell migration	Graupera et al. (2008)
Class I	Ocular angiogenesis	Sasore and Kennedy (2014)
PI3Kδ	Gestational diabetes	Wójcik et al. (2014)
PI3Kδ	Diabetic retinopathy	Wu et al. (2020), Liu et al. (2024)
Class IA	Microglial dependent retinal neovascularization	Chen et al. (2023)

Main upstream biochemical pathway involved in DR pathogenesis and progression is the VEGF/PI3K/AKT, although other growth factor signaling pathways cannot be excluded (Li et al., 2023). Other signaling pathways have been linked to PI3K activation in DR, among them, are enlisted also the advanced glycation end products receptor (RAGE) signaling (Shu et al., 2025) and the PI3K/AKT/HIF1α (Treins et al., 2002).

As regards the GPCRs/PI3K signaling that would be involved in diabetes and diabetic retinopathy, the number of combinations dramatically increases, spanning from catecholamines to peptides such as GLP-1, as evidenced by a recent review (Varney and Benovic, 2024). We thereby try to focus on PI3K isoforms, instead of the upstream signaling, due to the great number of ligands in advanced clinical trial phases, and specifically few studies focused on involvement of PI3K isoforms in DR pathogenesis and progression (Table 1).

Although expression and activity of p110β and p110δ has been detected in immortalized endothelial cells, it was shown that p110α was necessary for angiogenesis and cell migration upon VEGF-A stimulation (Graupera et al., 2008). However, it has been proven, that pan inhibition of class I PI3Ks was effective in inhibition of ocular angiogenesis in zebrafish, while a p110α inhibitor (A66, Selleckchem S2636) did not blocked ocular angiogenesis (Sasore and Kennedy, 2014). As regards pericytes (iBRB), it has been found that activity of PI3Kβ modulates pericyte maturation and vessel remodeling. In fact, early pericyte differentiation (low PI3Kβ signaling), induces endothelial cell quiescence; high PI3Kβ signaling is linked to pericyte immaturity which leads to vascular hyperplasia and inhibition of vascular remodeling (Figueiredo et al., 2020). These findings on microvascular pericytes, give hints about the angiogenesis and vascular remodeling during embryogenesis, but not during pathologic angiogenesis, such as in DR. Worthy of note, human retinal endothelial cells have been reported to express PI3Kδ, whose levels increase under hyperglycemic insult. Interestingly, Wu et al., 2020 have shown that PI3Kδ was not expressed in cone-like cell line 661W (Wu et al., 2020). Thereby, putative photoreceptor toxicity or associated resistance mechanisms, as recorded for anti-VEGF agents (Sitnilska et al., 2019) would not occur through PI3Kδ inhibition. In fact, Wu et al., 2020 have found that single dose intravitreal injection of a PI3Kδ inhibitor (idelalisib) did not affect photoreceptor function in mice pups. PI3Kδ inhibitor treatment, or genetic knock down of PI3Kδ in mice, resulted in inhibition of retinal angiogenesis in a mouse model of oxygen induced retinopathy (Wu et al., 2020).

Recently, it has been shown that within all PI3K isoforms the PI3Kδ was expressed in epiretinal membrane of patients with diabetic retinopathy (Liu et al., 2024). Epiretinal membrane is a consequence of retinal fibrosis. Interestingly, treatment with a PI3Kδ inhibitor (idelalisib) inhibited retinal fibrosis *in vitro* and *in vivo* (Liu et al., 2024). Retinal fibrosis is a secondary and irreversible complication of diabetic retinopathy, and one of the main pathways implicated in this pathological mechanism is the TGF-β1 pathway (Yanagisawa et al., 1993; Platania et al., 2018; Bonfiglio et al., 2020), also through the smad-independent signaling, such as the TGFβ1/PI3K/AKT (Daley et al., 2023; Tang et al., 2023; Hartmane and Mikazans, 2025).

Currently, available literature (Table 1) does not suggest involvement of PI3K isoforms, other than PI3Kδ, in diabetic retinopathy onset or progression. In fact, Wu et al., 2020 demonstrated, through genetic knock down in mice, that the PI3Kδ does not affect physiological vessel development, such as the PI3Kα and β, but is involved in angiogenesis only in pathological conditions (Wu et al., 2020).

**FIGURE 3:**
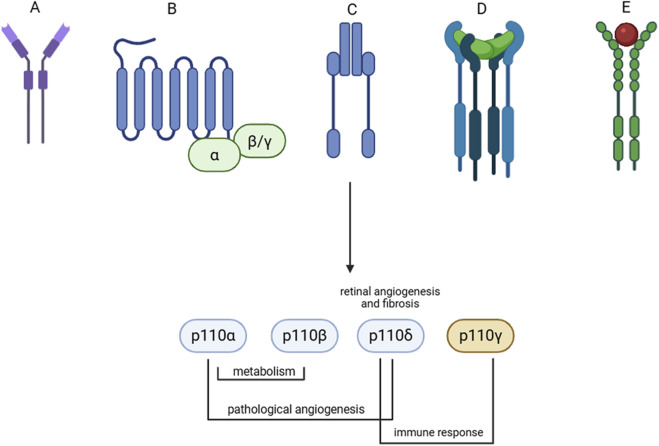
Upstream signaling to PI3K activation in diabetic retinopathy. **(A)** Advanced glycation end product receptor; **(B)** GPCRs; **(C)** Insulin receptor; **(D)** TGFβ1 receptor; **(E)** VEGF receptor. Class IA (blue) and class IB (orange) subunit, and involvement in pathological mechanism in diabetes mellitus and diabetic retinopathy (resume in Table 1).

Thereby, within all selective PI3K inhibitors already approved, PI3Kδ inhibitors would be good candidates for management of diabetic retinopathy specific pathogenic mechanisms (angiogenesis and fibrosis), mediated by different receptors as shown in Figure 3. As mentioned in previous paragraphs, PI3Kδ and PI3Kγ are mainly expressed in immune B and T-cells (Dawson, 1990; Lanahan et al., 2024). Involvement of immune response, promoted by lymphocytes and resident microglial cells, in chronic inflammation is detrimental for diabetic retinopathy progression (Bulu and Keser, 2025; Kinuthia et al., 2025). Specifically, a recent study highlighted that PI3K signaling in microglia promotes pathological retinal neovascularization (Chen et al., 2023).

An involvement of specific PI3K isoforms, other than PI3Kδ, in age-related macular degeneration, and other retinal proliferative diseases, up to now, cannot be excluded, since no studies have been retrieved about selective class I PI3K inhibition.

## 4 Class I PI3K inhibitors

We analyzed pharmacodynamic (PD) and pharmacokinetic (PK) aspects of selective inhibitors of different PI3Ks isoforms with focus on enzymes members of class IA as a treatment for human diseases: copanlisib, idelalisib, duvelisib, umbralisib, seletalisib, leniolisib (Table 2). These compounds have been approved, withdrawn after approval or have reached an advanced clinical trial step. Specifically, compalisib, idelalisib and duvelisib are currently approved for different forms of relapsed lymphoma (Cant et al., 2024).

**TABLE 2:** PD and PK of PI3K inhibitors.

​	​	​	​	IC_50_(nM)	​	​	Cl^+^	Vd^+^
Inhibitors	Indication	PI3Kα	PI3Kβ	PI3Kγ	PI3Kδ	γ/δ IC_50_ ratio	L/h	L
Copanlisib	Relapsed FL	0.5	3.7	6.4	0.7	9.14	24.8	871
Idelalisib	Relapsed CLL	820	565	89	2.5	35.59	14.9	23.9
Duvelisib	Relapsed or refractory CLL and SLL	1602	85	27	2.5	10.79	3.6–11.2	26–102
Umbralisib	Approval Withdrawn	>10,000	1116	1065	22	48.30	15.5	312
Seletalisib *	NCT02610543 NCT02303509 NCT02207595	active	active	280	12	23	1.7	60.9
Leniolisib	APDS	244	424	2230	11	204.08	4	28.5

FL, follicular lymphoma; CLL, chronic lymphocytic leukemia; SLL, small lymphocytic lymphoma. This table was partially adapted from references (Tommasini et al., 2024). Other data have been retrieved from annotated databases (^*^PubChem, ^+^DrugBank). IC_50_ = Inhibitory Concentration 50; Cl = clearance; Vd = distribution volume.

Umbralisib has been approved for follicular lymphoma and marginal zone lymphoma, but in 2022 the company voluntary withdrawn the drug for increased risk of death during the trial, in patients with chronic lymphocytic leukemia and small lymphocytic lymphoma (Cant et al., 2024). As regards as seletalisib, this compound has been evaluated in clinical trial for treatment of primary Sjögren’s syndrome, but the trial NCT02610543 has been terminated due to recruitment problems. Besides, trial termination, seletalisib has been found to improve symptoms in patients with Sjögren’s syndrome, with good tolerability (Juarez et al., 2021). Seletalisib has been also evaluated for safety and tolerability in healthy subjects and in patients with mild-to-moderated psoriasis (NCT02303509, NCT02207595) (Helmer et al., 2017). These two trials evidenced that seletalisib was well tolerated by recruited subjects, and no serious adverse effects have been recorded. Furthermore, seletalisib is currently evaluated for treatment of the rare immunodeficiency APDS (Diaz et al., 2020).

Within compounds reported in Table 2, the most potent PI3Kδ inhibitors are copanlisib < idelalisib < duvelisib. Copanlisib is a pan PI3K class I inhibitor, bearing nanomolar or subnanomolar activity over all PI3K class I enzymes. Idelalisib, duvelisib, umbralisib and seletalisib have comparable γ/δ IC50 ratio, while leniolisib has shown the highest selectivity (γ/δ IC50 ratio, 204.08) toward the PI3Kδ. Within all class I PI3K inhibitors evaluated in clinical trials, or approved for treatment of onco-hematological diseases, leniolisib has been shown to be well tolerated by patients with APDS syndrome. Leniolisib safety profile has been accounted to its mechanism of action: low potency but high selectivity for the mutated isoform PI3Kδ, which is mutated in APDS patients (Cant et al., 2024). Clearance (Cl, L/h) and the distribution volume (Vd, L) of inhibitors (Table 1) have been retrieved from the database DrugBank. As regards as leniolisib, its apparent Vd is 28.5 L suggesting a a peripheral distribution, for the oral administration root. Additionally, leniolisib has one of the lowest clearance levels in comparison to other inhibitors reported in Table 1. Thus, the safety and tolerability profile of leniolisib, on the basis of the data collected by treated APDS patients, can be attributed also to drug’s pharmacokinetic parameters, which avoid drug accumulation phenomena, then contributing to leniolisib high tolerability.

Pharmacokinetic properties, reported in Table 2, suggest a significant impact on safety and tolerability of PI3K inhibitors administered systemically. In ophthalmology, the main routes of drug administration are the ocular topical and intravitreal routes; the latter is the gold standard for management of posterior segment diseases, such as diabetic retinopathy. Intravitreal administration of drugs provides no uncertainty on ocular availability, and whenever ocular barriers are intact the systemic drug availability would be negligible (Bucolo et al., 2012). But several retinopathies, including diabetic retinopathy, are characterized by blood-retinal barrier breakdown (Hang et al., 2025). Additionally, topical ocular administration of drugs is characterized, by systemic drug absorption, that although with minor extent compared to other routes, would lead to systemic drug effects (Bucolo et al., 2012).

However, the intravitreal administration of drugs is an invasive and costly route, because it needs an inpatient setting. Topical ocular formulations, targeting the posterior segment of the eye, can take advantage of nanotechnology, providing an increased vitreo-retinal drug availability compared to traditional eye drop formulations (Bucolo et al., 2012; Platania et al., 2019; Cimino et al., 2024; Conti et al., 2025). Thereby, formulative studies on PI3Kδ inhibitors are worthy to be carried out, in order to further evaluate *in vivo* ocular bioavailability of drug eye drops, such as leniolisib.

## 5 Discovery of novel PI3Kδ selective inhibitors

Based on these evidences related to efficacy, tolerability and toxicity of PI3K class I inhibitors, and considering that PI3Kδ is an intriguing pharmacological target for management of diabetic retinopathy, next medicinal chemistry efforts should be focused on drug design (or repurposing), aimed at discovery new PI3K inhibitors with high γ/δ IC_50_ ratio. Integrated approaches, such as computer-aided drug design (CADD), would help to achieve this aim. CADD technologies such as homology modeling, pharmacophore modeling, quantitative conformational relationships, molecular docking, molecular dynamics simulation, binding free energy prediction, and high-throughput virtual screening, can effectively improve the efficiency of new drug discovery (Wu et al., 2024). The CADD strategy is based on investigating structure-based drug design (SBDD) and ligand-based drug design (LBDD) (Yu and Mackerell, 2017).

**FIGURE 4:**
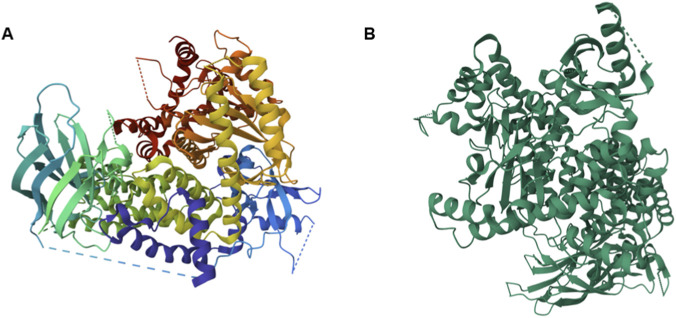
PI3K 3D structures. **(A)** Crystal structures of human Phosphatidylinositol-4,5-bisphosphate 3-kinase catalytic subunit gamma isoform (PDB: 5G2N) (Scott et al., 2016). **(B)** Crystal structures of human Phosphatidylinositol-4,5-bisphosphate 3-kinase catalytic subunit delta isoform (PDB: 4GB9) (Murray et al., 2012).

The information concerning protein structure can be collected through databases, such as the Protein Data Bank (PDB), which allows to analyze the 3D structure of proteins (Figure 4) and nucleic acids deposited from crystallographic images or NMR spectroscopy (https://www.rcsb.org/).

Through virtual screening, it is possible to investigate and optimize the structure of PI3K inhibitors for oncological treatment. In fact, a new series of selective PI3Kδ inhibitors has recently been described using a combined ligand- and receptor-based virtual screening workflow. This has led to compounds with improved permeability and pharmacokinetic profile (Fradera et al., 2021).

**FIGURE 5:**
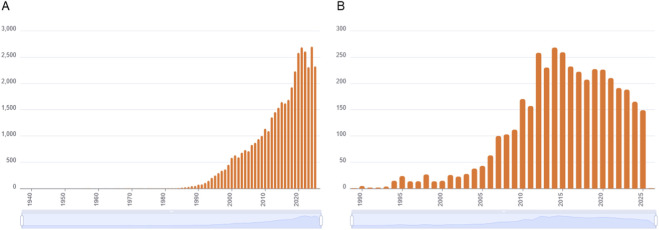
Entries about PI3K inhibitors. **(A)** Number of publications containing the keywords “PI3K″ or “phosphoinositide 3-kinase” in the title. **(B)** Number of publications containing the keywords “PI3K″ or “phosphoinositide 3-kinase” and the term “inhibitor” in the title. Source: CAS SciFinder, October 2025.

Overall, the clinical opportunities linked to PI3K inhibition have generated great interest in the discovery and development of inhibitors and, to date, more than 3800 studies, including about 1000 patent. The highest number of publications was recorded between 2012 and 2015 (Figure 5); in this period the Food and Drug Administration approved the first PI3K inhibitor, idelalisib, for relapsed chronic lymphocytic leukemia, follicular lymphoma and small lymphocytic lymphoma.

The Binding Database (Gilson et al., 2016) reports on more than 35K compounds tested on the class I “Phosphatidylinositol 3-kinase regulatory subunit alpha/4,5-bisphosphate 3-kinase” targets. Most of the studies concern inhibitors of the PI3Kα and PI3Kδ isoforms with, respectively, 550 works and 11K inhibitors tested for the first and 472 works and 17K inhibitors tested for the latter isoform. The experimental data reported are extremely heterogeneous both in terms of the parameter that defines the activity of the inhibitors (expressed as K_i_, IC_50_, K_D_ or EC_50_) and the type of test used to measure the inhibitory activity.

This huge amount of data has been the subject of a limited number of quantitative structure-activity relationship (QSAR) studies aimed at designing new selective PI3K kinase inhibitors. Most of these studies deal with the design of PI3Kα isoform inhibitors or dual PI3Kα/mTOR inhibitors. On the contrary few reports concern Pan (Safavi-Sohi and Ghasemi, 2013; Zhu et al., 2024) inhibitors and only recently studies have been focused on selective PI3Kδ inhibitors.

### 5.1 QSAR studies on PI3Kα inhibitors

The first 3D-QSAR analysis of phosphoinositide 3-kinase p110α inhibitors has been proposed in 2008 (Frédérick and Denny, 2008). This study combined homology modeling and 3D-QSAR, based on comparative molecular field analysis (CoMFA) and comparative molecular similarity indices analysis (CoMSIA) methods, to generate an integrated interaction model able to correlate the chemical structures of 25 imidazo [1,2-*a*]pyridine p110α selective inhibitors with their biological activities. The study highlights that, to selectively target p110α, the central (hetero)aromatic scaffold bearing an H-bond acceptor should be substituted on one side with a small lipophilic group and on the other side with two H-bond acceptors, in order to interact with the ATP-binding site and with residues highly specific to p110α.

A larger training set was employed to develop a different model in 2010 (Li et al., 2010): the structures of the 66 compounds and their observed activity (pIC50) against PI3K p110α were all originated from the same laboratory (Hayakawa et al., 2006; Hayakawa et al., 2007b; Hayakawa et al., 2007a; Hayakawa et al., 2007c). The dataset includes 24 4-morpholino-2-phenylquinazolines derivatives, 16 pyrido [3′,2′:4,5]furo [3,2-d] pyrimidine derivatives, 12 imidazo [1,2-a]pyridine derivatives and 13 sulfonylhydrazone substituted imidazo [1,2-a]pyridines and LY294002 (Hayakawa et al., 2006) as lead compound. This ligand-based 3D-QSAR model identified a five-point pharmacophore with three hydrogen bond acceptors (A), one lipophilic/hydrophobic group (H), and one aromatic ring (R). The selected model has good statistical significance and good predictive ability.

The comparative molecular field analysis (CoMFA) and comparative molecular similarity indices analysis (CoMSIA) methods were performed in 2012 (Wang et al., 2012) to build predictive 3D-QSAR models, starting from a database of 61 benzothiazoles PI3Kα/mTOR dual inhibitors (D’Angelo et al., 2011). This analysis identified regions where bulky groups, negative/positive charges, electron-donating and electron-withdrawing groups, hydrophilic and hydrophobic substituents, H-bond acceptor groups increase or decrease the activity. The former dataset, comprising 61 benzothiazole PI3Kα/mTOR dual inhibitors, was also employed to develop descriptor and pharmacophore-based QSAR models (Bharate et al., 2013). The most relevant descriptors of the descriptor-based model, developed for PI3Kα activity by stepwise multiple regression analysis (MRA) and partial least square (PLS) methods are the moment of inertia one size, the ChiV5 path index and the number of H-bond donors. All three descriptors are contributing positively but to a different extent to the variation in pKi values. The same dataset was used to develop the pharmacophore-based 3D-QSAR model which comprises two H-bond acceptor, one H-bond donor, and two aromatic moieties. Interestingly, this model indicates the importance of an additional SO_2_ group (making sulfonamide linkage) for a relevant increase of the activity.

A different class of compounds, namely, 3-pyridinyl heterocyclic derivatives (Stec et al., 2011; Nishimura et al., 2011), was used to build 3D-QSAR models (based ligand and receptor alignment) to design and optimize (PI3Kα/mTOR) dual inhibitors using five different fields: steric, electrostatic, hydrophobic, hydrogen bond donor and acceptor fields (Yang et al., 2013). Ten novel derivatives with high predicted activities were designed using these 3D-QSAR models, but no further experimental data support the models.

A data set of 49 imidazo [1,2-a]pyrazine inhibitors of PI3Kα (Martínez González et al., 2012a; 2012b) was used for the development of atom-based 3D-QSAR model (Chadha et al., 2014). The analysis suggested that the selected scaffold can be substituted with a hydrophobic (-Cl,-Br, -F) group while the pyrimidine ring can be substituted with both a hydrophobic group (methyl or ethyl group) and hydrogen bond donor group (i.e., NH_2_, OH, CONH_2_). The authors reported also the activities of the seven best designed molecules, but we have not retrieved experimental data that support these models.

Atom-based QSAR models (Takeda et al., 2016) were also developed using 40 compounds based on the two-oxatriazine or Bis(morpholino-1,3,5-triazine) scaffold reported as highly potent PI3Kα/mTOR dual inhibitors (Venkatesan et al., 2010; Dehnhardt et al., 2011). The most active compounds for both enzymes showed hydrophobic/non-polar and electron-withdrawing effects at both ends of the compound structures including a morpholine and an amine moiety contributing to the inhibition activity. These data suggested that PI3Kα and mTOR shared the same mechanism of the inhibition and highlighted the slight difference of the shape of the binding site between PI3Kα and mTOR for the design of selective inhibitors.

Dual inhibition of PI3K and mTOR is the target of a 3D-QSAR pharmacophore modeling study (Oluić et al., 2017) performed using a dataset of 85 pyrazolopirimidine analogs as dual PI3K/mTOR inhibitors (Verheijen et al., 2009; Zask et al., 2009). The study revealed that creation of an adequate polar surface area around the molecule is important for the mTOR/PI3K inhibitory activity. New compounds were designed by structural modification of the most active compounds, through the addition of H-bond donors and H-bond acceptors, and 15 new dual inhibitors with enhanced predicted activity and good drug-like properties were selected after ADMET profiling.

A series of quinoline derivatives (Lv et al., 2015; Zhang et al., 2017a; 2017b), namely, N-(5-(quinolin-6-yl)pyridin-3-yl)benzenesulfonamides, 4-alkynyl-quinoline and 3-amidoquinoline derivatives as PI3K/mTOR dual inhibitors, were used in one of the most recent atom-based QSAR model (Peddi et al., 2018). The authors developed a five-point pharmacophore hypothesis with one hydrogen bond acceptor (A), one hydrogen bond donor (D), one hydrophobic group (H), and two aromatic rings (R); however, the presence of additional hydrophobic and H-bond acceptor features is beneficial for the activity. The overall results of this study revealed that compounds possessing indole and benzothiazole moieties can act as potent inhibitors against PI3Kα and, more interestingly, that even cyclic compounds can act as good inhibitors against PI3Kα on par with linear compounds.

To develop new quinazoline derivatives as PI3Kα inhibitor, thirty-one heteroaromatic derivatives were utilized in a QSAR model that was further combined with molecular docking, and molecular dynamics studies (Arba et al., 2018). The model was used to design a novel compound which has lower predicted IC50 than the previous lead compound, but no experimental validation of the result has been proposed.

A training set of 18 ATP-competitive PI3Kα inhibitors, derived from the ChEMBL (Bento et al., 2014) and BindingDB (Gilson et al., 2016), was used to generate a 3D-QSAR pharmacophore models based on hydrogen-bond acceptor (HBA), hydrogen bond donor (HBD), ring-aromatic (RA), and hydrophobic (HY) features (Yu et al., 2018). The best pharmacophore hypothesis, consisting of a HBD and two RA features, was able to predict the activities of the training set within one order of magnitude and was used to search for novel ATP-competitive PI3Kα inhibitors from SPECS database. Visual analysis of the docking poses and interactions between the ligands and the receptor allowed to identify 79 compounds for the bio-assay. Notably, with this screening protocol were discovered 10 new PI3Kα inhibitors with IC_50_ values ranging 0.44–31.25 μM.

### 5.2 QSAR studies on PI3Kγ inhibitors

The significant induced fit flexibility observed for PI3Kγ and the lack of adequate ligand-based computer-aided drug design studies for this isoform, led in 2014 to the development of a ligand-based three-dimensional (3D) pharmacophore(s) integrated within self-consistent QSAR model (Taha et al., 2014). This approach, chosen to limit the drawbacks of structure-based techniques, employs a total of 78 compounds with excellent 3D diversity and continuous bioactivity spread in a classical QSAR analysis to search for the best combination of pharmacophore(s) and 2D descriptors capable of explaining bioactivity variation. The authors identified two mergeable pharmacophoric models in the two different QSAR equations; this suggested the existence of combined binding mode accessible to ligands within the PI3Kγ binding pocket. It is noteworthy that the QSAR models were experimentally validated via an *in silico* screening of 110 NCI available compounds. The highest-ranking hits were evaluated *in vitro* against human PI3Kγ and this procedure allowed to identify 19 NCI hits with nanomolar to low micromolar potencies.

In 2019 a series of 31 potent selective PI3Kγ inhibitors was selected for a systematic computational analysis, combining 3D-QSAR, molecular docking, molecular dynamic (MD) simulations, free energy calculations and decomposition (Li et al., 2019). The information acquired from this extensive computational analysis were used to design ten derivatives and some of them show potent bioactivity profile confirming the possibility to develop potent PI3Kγ inhibitors through computational modeling. A similar approach was employed also in 2021 with a series of 39 isoindolinone-based inhibitors (Ghosh et al., 2021): based on the QSAR analysis, the authors designed 14 new P13Kγ inhibitors some of which exhibited higher pIC50 values than the data set compounds and obtained a reasonable statistical score in synthetic accessibility and ADME/Tox prediction.

A large set of 245 potent and selective isoform specific PI3Kγ inhibitors was used for a QSAR analysis using an artificial neural network and multivariate linear regression in 2022 (Sadeghi et al., 2022). The authors highlighted a direct relationship between the polarity of the compounds and their inhibitory activity: the type and coefficients of the model descriptors show that by increasing the electronegative atoms (N, O, or F) in the structures, their inhibitory activity is enhanced. Moreover, hydrophobic substituents such as bulky groups and long carbon chain substitution weaken the inhibitory activity of the molecules used as inhibitors of PI3Kγ enzyme while polar regions strengthen it.

### 5.3 QSAR studies on PI3Kδ inhibitors

QSAR studies on selective inhibitors for PI3Kδ are certainly less numerous and more recent.

The structure and activity of a series of quinazolinone scaffold-based PI3Kδ inhibitors, sharing the core structure of idelalisib and duvelisib, have been reported in 2017 (Peng et al., 2017). A set of 37 quinazolinone derivatives bearing a 2,4,6-triaminopyrimidine motif were selected for 3D-QSAR models constructed by comparative molecular field analysis (CoMFA) and comparative molecular similarity indices analysis (CoMSIA) methods; the partial least squares (PLS) analysis correlated the CoMFA, Topomer CoMFA and CoMSIA descriptors to the biological activity data revealing that electrostatic property contributed more for binding affinity of PI3Kδ inhibitor as compared to steric property. Molecular docking, pharmacophore model, and molecular dynamics simulation were used to analyze: i) the influence of pharmacophore groups on the inhibitory activity, ii) the binding patterns of ligands with PI3Kδ receptor and iii) the dynamics behavior of the ligand and obtain more detailed ligand–protein interaction information. Among the ten quinazolinone analogs designed, two compound showed better predicted activity and binding affinity with PI3Kδ; but the results have not been experimentally validated.

The need for better and safer PI3Kδ inhibitors led in 2019 to a QSAR-guided approach to define the best combination of crystallographic pharmacophores and physicochemical descriptors that explain binding affinity variation for a list of PI3Kδ inhibitors (Al-Sha’er et al., 2019). The authors collected the structures of 79 PI3Kδ inhibitors (Lin et al., 2012; Murray et al., 2012; Barlaam et al., 2015; Bui et al., 2015) and then implemented the genetic function algorithm and multiple linear regression analyses to select the optimal combination of pharmacophoric models and other physicochemical descriptors capable of self-consistent and predictive QSAR model. Only one pharmacophore was used in the QSAR model, suggesting that ligands bind into PI3Kδ *via* one major binding mode that involves for the selected lead: i) hydrogen bond acceptor/donor features anchoring the purine N/NH of the complexed ligand with the peptidic N-H and C=O of Val828 and Glu826; ii) hydrogen bonding interaction connecting the purine ring of the ligand with the phenolic hydroxy of Tyr813 *via* two bridging water molecules; iii) only limited hydrophobic binding interactions, namely, stacking the ligand’s chlorobenzene fragment against the indole of Trp760 and the hydrophobic side chain of Thr750. Interestingly, the ligand/PI3Kδ affinity is inversely correlated with ligands' negative charges while being directly proportional to the number of rotatable bonds of respective inhibitors. The results were used to screen the National Cancer Institute (NCI) database for new PI3Kδ inhibitors allowing to define several hits with low micromolar IC_50_ values.

The same authors used a similar approach in 2023 (Al-Sha’er et al., 2023) when the collected compounds were divided into four subsets based on four envisaged binding modes to select the binding hypotheses representative of ligand binding within PI3Kδ. The genetic function algorithm (GFA)-based QSAR modelling was used to select optimal combination of pharmacophore(s) and other 2D descriptors. However, two pharmacophores appear in the final QSAR equation suggesting that they represent discrete binding modalities that ligands can assume within the binding pocket of PI3Kδ. The central aromatic ring feature compares with π-stacking interaction anchoring the ligand to the phenol side chain of Tyr813 while the hydrogen bonding interaction locked the aromatic nitrogen of the ligand’s purine ring with the peptidic NH of Val828; two hydrophobic features correlates with hydrophobic interactions suggested by the close proximity to the indole of Trp760 and the hydrophobic side chain of Thr750 of binding pocket but extra aromaticity or unsaturation renders the molecules too rigid to fit in the binding pocket. They used the QSAR model to identify new PI3Kδ inhibitors of novel chemotype: the *in silico* screening yielded ten NCI hits with micromolar potencies, seven of which capable of inhibiting A549 alveolar lung cancer cells.

## 6 AI and machine learning approaches for discovery of PI3K selective inhibitors

Recently, machine learning models have been integrated to predictive modeling in drug discovery projects. This innovative approach, different from the QSAR studies described so far, generated a test system for predicting isoform selectivity of phosphoinositide 3-kinase inhibitor using a new methodology termed MolAnchor (Lamens and Bajorath, 2025). This led to identification of inhibitors with different isoform selectivity, well-defined and recurrent structural fragments.

**FIGURE 6:**
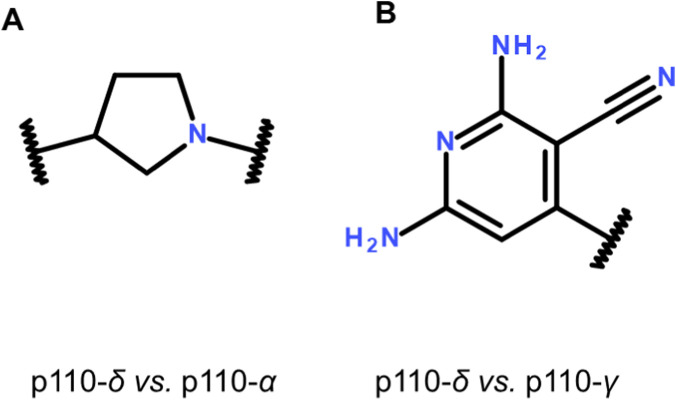
PI3K inhibitors selectivity. Most frequently substructure of selective p110-δ inhibitors. **(A)** δ vs. α; (B) δ vs.γ.

To generate PIK3 isoform selectivity data sets, human protein kinase inhibitors were extracted from BindingDB (Gilson et al., 2016) and CHEMBL (Bento et al., 2014); each dataset consisted of varying numbers of (∼1000) nonselective and (16–264) isoform selective inhibitors. The authors classified Inhibitors (for which numerically specified IC_50_, *K*i, or *K*d potency values of at least 10 *μ*M were available) as nonselective if their potency difference for two isoforms was at most 10-fold, and as selective for one isoform over another if their potency difference was at least 100-fold. For each selectivity data set and class of isoform-selective inhibitors, the authors identified the substructure most frequently occurring across independent prediction trials, namely, a pyrrolidine ring for selective p110-*δ* inhibitors from the (p110-*δ*-p110-*α*) set and 4-diaminopyrimidine-5-carbonitrile for selective p110-*δ* inhibitors from the (p110-*δ*-p110-*γ*) (Figure 6).

Machine learning was also employed to conduct a virtual screening against PI3K of the cardiac glycosides of Vernonia amygdalina (Syauqy Tafrihani et al., 2025). The authors selected 6079 PIK3CA inhibitor compounds with standard IC_50_ values sourced from ChEMBL (Bento et al., 2014) to obtain the training dataset and the optimal predictive model was used to screen cardiac glycosides obtained from the ethyl acetate fraction from the Vernonia amygdalina. The three compounds with the highest prediction scores, namely, Vernoamyosides A (VG-1), Vernoniamyosides D (VG-8), and Vernoniosides A4 (VG-10), were then subjected to molecular docking showing stronger and more stable interactions with the PIK3CA receptor compared to alpelisib. Further molecular dynamics simulations indicated that compounds VG-10 formed the most stable complex when compared to the other compounds.

## 7 Conclusion

PI3K signaling involves several physiological cellular functions and pathological mechanisms, in onco-hematological, inflammatory, and rare immunological diseases. Class IA PI3Ks are activated downstream to receptor tyrosine kinases such as VEGF receptors; unbalanced signaling leads to angiogenesis in proliferative retinal diseases, such as diabetic retinopathy and age-related macular degenerations. Specifically, involvement of PI3Kδ isoform has been found in diabetic retinopathy and in retinal fibrosis, a complication of diabetic retinopathy. Retrieval of PD profiles of already approved or investigational class I PI3K inhibitors, evidenced that within all PI3K inhibitors, leniolisib has the highest selectivity toward the PI3Kδ isoform over the PI3Kγ. Indeed R&D programs that would focus on PI3Kδ selective inhibitors would burst the research and development of new safe and effective drugs for treatment of diabetic retinopathy.
